# Rectal/urinary toxicity after hypofractionated vs conventional radiotherapy in low/intermediate risk localized prostate cancer: systematic review and meta analysis

**DOI:** 10.18632/oncotarget.14798

**Published:** 2017-01-22

**Authors:** Rossella Di Franco, Valentina Borzillo, Vincenzo Ravo, Gianluca Ametrano, Sara Falivene, Fabrizio Cammarota, Sabrina Rossetti, Francesco Jacopo Romano, Carmine D’Aniello, Carla Cavaliere, Gelsomina Iovane, Raffaele Piscitelli, Massimiliano Berretta, Paolo Muto, Gaetano Facchini

**Affiliations:** ^1^ Progetto ONCONET2.0 Linea progettuale 14 per limplementazione della prevenzione e diagnosi precoce del tumore alla prostata e testicolo Regione Campania, Italy; ^2^ Radiation Oncology, Istituto Nazionale per lo Studio e la Cura dei Tumori Fondazione Giovanni Pascale IRCCS, Napoli, Italy; ^3^ Division of Medical Oncology, A.O.R.N. dei COLLI Ospedali Monaldi-Cotugno-CTO, Napoli; ^4^ Department of Onco-Ematology Medical Oncology, S.G. Moscati Hospital of Taranto, Taranto, Italy; ^5^ Division of Medical Oncology, Department of Uro-Gynaecological Oncology, Istituto Nazionale Tumori Fondazione G. Pascale - IRCCS, Naples, Italy; ^6^ Department of Medical Oncology, CRO Aviano, National Cancer Institute, Aviano, Italy

**Keywords:** prostate cancer, radiotherapy, toxicity, meta-analysis, review

## Abstract

**Purpose:**

The aim of this review was to compare radiation toxicity in Localized Prostate Cancer (LPC) patients who underwent conventional fractionation (CV), hypofractionated (HYPO) or extreme hypofractionated (eHYPO) radiotherapy. We analyzed the impact of technological innovation on the management of prostate cancer, attempting to make a meta-analysis of randomized trials.

**Methods:**

PubMed database has been explored for studies concerning acute and late urinary/gastrointestinal toxicity in low/intermediate risk LPC patients after receiving radiotherapy. Studies were then gathered into 5 groups: detected acute and chronic toxicity data from phase II non randomized trials were analyzed and Odds Ratio (OR) was calculated by comparing the number of patients with G0-1 toxicity and those with toxicity > G2 in the studied groups. A meta-analysis of prospective randomized trials was also carried out.

**Results:**

The initial search yielded 575 results, but only 32 manuscripts met all eligibility requirements: in terms of radiation-induced side effects, such as gastrointestinal and genitourinary acute and late toxicity, hypofractionated 3DCRT seemed to be more advantageous than 3DCRT with conventional fractionation as well as IMRT with conventional fractionation compared to 3DCRT with conventional fractionation; furthermore, IMRT hypofractionated technique appeared more advantageous than IMRT with conventional fractionation in late toxicities. Randomized trials meta-analysis disclosed an advantage in terms of acute gastrointestinal and late genitourinary toxicity for Hypofractionated schemes.

**Conclusions:**

Although our analysis pointed out a more favorable toxicity profile in terms of gastrointestinal acute side effects of conventional radiotherapy schemes compared to hypofractionated ones, prospective randomized trials are needed to better understand the real incidence of rectal and urinary toxicity in patients receiving radiotherapy for localized prostate cancer.

## INTRODUCTION

Prostate cancer is one of the most frequent tumors affecting men in the world: external beam radiotherapy (EBRT) is a standard treatment modality for localized and locally advanced prostate cancer [1, 2].

Modern technologies, predictive biomarkers of response to a given therapy, potential new targets for biological therapy and advanced knowledge of radiobiology have changed the approach to prostate cancer radiotherapy [3–5].

Many publications suggest that prostate cancer has a low α/β ratio (ratio between “intrinsic radiosensitivity” and “reparative capacity”), compare to healthy tissues [1,2,6] with notable therapeutic implications [6–8]. In the treatment of prostate cancer, we can’t diseregard that organs at risk (OARs), as rectum or bladder, have an estimated α/β ratio of 3-5 Gy for late toxic effects and 10 Gy for acute toxicity, whereby prostate cancer cells are more responsive to a larger fraction size, with a clear therapeutic gain [9].

Five large randomized trials demonstrated that increasing the dose to 74-80 Gray (Gy), fractionated in standard 1.8-2 Gy, results in an improved biochemical recurrence-free and disease free survival [10–14]. Treatments planned with dose escalation and hypofractionation have been made possible thanks to the evolution of radiation therapy techniques. Further advances in radiation delivery techniques, such as intensity modulated radiation therapy (IMRT) and volumetric modulated arc therapy (VMAT), led to a greater sparing of adjacent normal tissue and consequent reduced toxicity. Significant reduction of margins around the prostate, and thus irradiated normal tissue volume, has been achieved by the use of daily cone-beam computed tomography imaging prior to each treatment delivery [15].

Radiation techniques for localized prostate cancer involve both external beam radiation and brachytherapy. External beam techniques include IMRT, VMAT and helical tomotherapy. Extremely hypofractionated (eHYPO) radiation regimens, consisting of 5 treatment sessions or less, have also been investigated. Current approach in prostate HYPO and eHYPO radiotherapy trials utilizes a simultaneous integrated boost (SIB) technique to deliver higher dose to dominant intraprostatic lesions, while still delivering an adequate lower dose to the whole prostate. Cyber-knife system (CK) makes use of fiducial markers, allowing to follow the target organ and reduce the irradiation volume.

A second dose escalation strategy involves proton therapy: protons are charged particles that deposit a higher proportion of energy toward the end of their travel path in a tissue and little to no energy beyond. A very steep dose gradient can be created to minimize dose spill into adjacent tissues, compared to photon therapy. At the moment, the little experience of large proton centers do not show superiority in disease control or toxicity for proton therapy compared to photon therapy [16–18].

Patient selection is fundamental for the choice of treatment, which must consider various aspects in order to define the risk class. Based on pre-treatment prognostic parameters, several systems have been proposed to stratify prostate cancer into differing risk groups. In 2010, the seventh edition of the AJCC (American Joint Committee on Cancer) Staging Manual [19] added Gleason score and PSA to the TNM staging system, making this stage grouping roughly comparable to D’Amico’s and NCCN ones, with notable differences between intermediate- and high-risk groups. NCCN (National Comprehensive Cancer Network) also added “very low-risk” and “very high-risk” categories. Nearly 50% of patients diagnosed with prostate cancer fall in prognostic AJCC Stage I, which includes patients with a clinical stage of T1-T2a, PSA≤10 and Gleason ≤ 6 [20].

Aim of this review is to compare radiation toxicity data detected in conventional fractionation (CV), hypofractionated (HYPO) and extreme hypofractionated (eHYPO) studies, based on different techniques used.

## RESULTS

### Study selection

**Figure 1:**
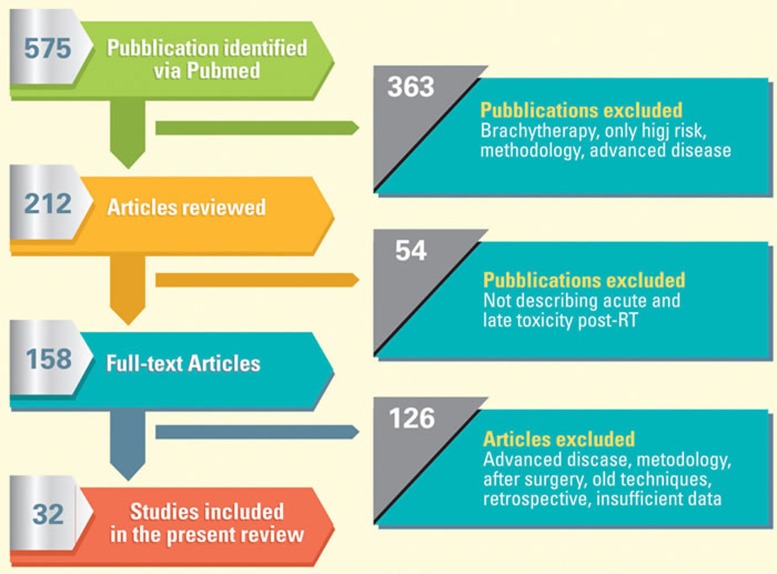
Analysis flow-chart of published literature evaluating the acute and late genitourinary and gastrointestinal toxicity following prostate radiation therapy The initial search yielded 575 results, but only 32 manuscripts met all eligibility requirements and were included in this report.

Search results are summarized in Figure 1. The initial search yielded 575 results. 363 publications were excluded (brachytherapy, only high risk, only methodology, advanced disease), which dropped down the initial number to 212. These articles were reviewed and 54 studies, which did not evaluate both acute and chronic toxicity genitourinary GU and gastrointestinal GI, were removed. 158 full-text articles were finally evaluated but further 126 studies were discarded because assessing after-surgery treatments, old techniques, retrospective studies or had few data. In total 32 manuscripts met all eligibility requirements and were included in this report.

Among the selected articles, three groups were obtained. Group I gathers 11 articles regarding 3DCRT treatments, including 5 with conventional fractionation (3 with EBRT, 2 with Protons), 4 articles with hypofractionated radiotherapy (1 randomized) and 2 articles concerning mixed techniques application (3DCRT and SBRT with Cyberknife system) (Table 1). The second group includes 11 studies of treatments with IMRT techniques (4 with conventional fractionation and 4 with hypofractionated, 3 randomized studies hypofractionated/conventional) (Table 2). The third group includes 10 studies of extreme hypofractionated treatment (4 used Linac, 6 Cyberknife system) (Table 3). Table 4 shows mean of the percentage for toxicity G2 and > G3 detected in the different study groups.

**Table 1:** Summary of trials on 3DCRT treatments with conventional and hypofractionated treatment

Author	Year (No. pt)	Type of Study	FU (median)	Risk groups (L/I/H) %	Technique	RT (Total dose/n.fz) (daily fz)	[EQ D2] a/b 1,5	ADT	Acute GU Toxicity %	Acute GI Toxicity %	Late GU Toxicity %	Late GI Toxicity %	Toxicity (Criteria)
**Schmid et al**	2012 (123)	Prospective phase II	74 M	L:35;I:34;H:30	3DCRT CV	70Gy/35 (2)	[70 Gy]	135(+) 43(-)	>G2:22	>G2:15	> G2:23	>G2:19	EORTC RTOG
	(55)					74Gy/37(2)	[74 Gy]						
**Michalsky et al**	2005 (119)	Prospective Phase I-II	6M	L:54% I: 46%.	3DCRT CV	78 Gy/39 (2) prostate	[78Gy]	43(+) 176(-)	G0-G1:60;: G2:36; G3:4%	G0-G1:60;: G2:36; G3:4%	G1:25; G2:14; G3: 3	G1:20; G2:19; G3: 2	RTOG
	(100)					78 Gy/39 (2) Prostate+vs			G0-G1:53;: G2:45; G3:2%	G0-G1:53;: G2:45; G3:2%	G1:19; G2:20; G3: 4	G1:25; G2:17; G3: 3; G4:2	
**Zietman et al**	2010 (197)	Prospective randomized	105 M	70,2 Gy L:57;I:38;H:5	3DCRT +boost protoni CV	70.2-79,2 Gy 50.4Gy/ (1.8) Boost 19.8GyE/11(1.8)	[70.2 Gy]+[19.8Gy]	393(-)	[70,2 Gy] G1:37;G2:51;G3:3	[70,2 Gy] G1:39;G2:44;G3:1	[70,2 Gy] G1:42;G2:22;G3:2	[70,2 Gy] G1:35;G2:13	RTOG
	(197)			79,2Gy L:59;I:35;H:4		70.2-79,2 Gy 50.4Gy/ (1.8) Boost 28.8GyE/16 (1.8)	[79.2 Gy]+[28.8Gy]		[79,2Gy] G1:29;G2:60;G3:2;G4:1	[79,2Gy] G1:26;G2:63;G3:1	[79,2Gy] G1:45;G2:27;G3:2	[79,2Gy] G1:41;G2:24;G3:1	
**Coen et al**	2011 (85)	Prospective Phase II	31,6 M	L-I:100	Protoni CV	78-79 Gy/39-40(2)	[82 Gy]	85(-)	G1:51;G2:13;G3:1	G1:19;G2:1	G1:33;G2:26; G3:11;G4:2	G1:33;G2:26;G3:11;G4:2	EORTC RTOG
**Nihei et al**	2011 (151)	Prospective phase II	43,4 M	L:77; I:74	Protoni CV	L: 74Gy/37 (2) pros I: boost 24Gy pros after 50Gy prostate+vs	[74 Gy] [24 Gy] [50 Gy]	42(+) 109(-)	G1:58;G2:12	G1:10;G2:1	G1:6;G2:5;G3:1	G1:18;G2:3	CTC
**White et al**	2015 (90)	Prospective	36 M	L:11;I:38;H:50	3DCRT Hypo	57Gy/17 (3)	[73,3 Gy]	71(+) 19(-)	G1:58,6;G2:10;G3:1,1	G1:75,6	G1:47,3;G2:2,4	G1:40;G2:9,3;G3:4,7	CTCAE
**Tramacere et al**	2015 (97)	Prospective Phase II	39M	L:19;I:41;H:40	3DCRT Hypo	62Gy/20 (3,1)	[81,5Gy]	94(+)	G1:43;G2:21;G3:3	G1:18;G2:15	G1:11;G2:5;G3:3	G1:2;G2:10;G3:1	RTOG EORTC
**Jereczek-Fossa et al**	2013 (337)	Prospective	19 M	L:40.9;I:43.32;H:14.2	3DCRT Hypo/IGRT	70Gy/28 (2,5)	[74 Gy] [24 Gy] [50 Gy]	243(+) 94(-)	>G2:35;>G3:6.2	>G2:11.3;>G3:1.2	>G2:10.4;>G3:1.6	>G2:7.5;>G3:1.3	RTOG
**Lukka et al**	2005 (470)	Prospecitve randomized	67 M	/	3DCRT CV	66Gy/33(2)	[66Gy]	/	G3-G4:4.9	G3-G4:2.6	G3-G4:1.9	G3-G4:1.3	NCIC
	(466)				3DCRT Hypo	52.5Gy/20(2.62)	[61.9Gy]		G3-G4:8.6	G3-G4:4.1	G3-G4:1.9	G3-G4:1.3	
Mixed Techniques													
**Pontoriero et al**	2016 (26)	Retrospective	21,5 M	L:35;I:46;H:19	EBRT + CK(boost)	38Gy/4(9.5)vs 9,5Gy/2(4.75) + 46Gy/23(2)	[119.4 Gy] [17 Gy] [46 Gy]	16(+) 20(-)	G1:46;G2:4	G1:23;G2:8	G1:12;G2:4	G1:4	CTCAE
**Katz et al**	2010 (75)	Prospective	33 M	I:57;H:43	EBRT + CK(boost)	21Gy/7(3) boost 18-19,5/2(9-9.75)	[27 Gy] [54 Gy] [62.7 Gy]	36(+) 39(-)	G1:83,6;G2:6,8	G1:78,1;G2:6,7	G1:5,5;G2:4,1;G3:1,4	G1:5,5;G2:8,2	RTOG

Risk classes, technique used, total dose, type of fractionation, equivalent dose, acute and late gastrointestinal and genitourinary toxicity. Abbreviations: FU (Follow up); Risk groups: L (Low), I (Intermediate), H (High); CV (conventional); Hypo (Hypofractionated); EQ (Equivalent Dose); CK (Cyberknife); EBRT (External Beam Radiation Therapy); IGRT (Image Guided Radiotherapy); GU (Genitourinary); GI (Gastrointestinal); ADT (androgen deprivation therapy).

**Table 2:** Summary of trials on treatment with IMRT technique

Author	Year (No. pt)	Type of Study	FU (median)	Risk groups (L/I/H) %	Tecnique	RT (Total dose/n.fz) (daily fz)	[EQ D2] a/b 1,5	ADT	Acute GU Toxicity %	Acute GI Toxicity %	Late GU Toxicity %	Late GI Toxicity %	Toxicity (Criteria)
**Fang et al**	2015 (213)	Prospective	24 M	L:53;I:37;H:7,4	IMRT CV	79,2Gy/44(1.8)	[74.7Gy]	IMRT 66 (-);27(+)	[IMRT] G0-G1:71,3; G2-G3:28,7	[IMRT] G0/G1:86,2;G2/G3:13,8	[IMRT] > G2:18,3	[IMRT] > G2:10,8	CTCAE
	(181)				PBT CV			PBT 79 (-);15(+)	[PBT] G0-G1:78,7;G2-G3:21,3	[PBT] G0-G1:95,7;G2-G3:4,3	[PBT] > G2:12,8	[PBT] > G2:12,8	
**Goineau et al**	2013 (38)	Prospective	54 M	L:18;I:60;H:32	IMRT CV	76Gy/38(2)	[76Gy]	23(+) 15(-)	G1:36,8;G2:5,3;G3:2,6	G1:23,7;G2:5,3 G30	G1:34,2;G2:5,3;G3:5,3	G1:23,7;G2:15,8	CTCAE
**Marchand et al**	2010 (55)	Prospective	18 M	L:18; I:60,2; H:21,8	IMRT CV	76Gy/38(2)	[76Gy]	25(+) 30(-)	G2:38;G3:2	G2:13	G2:15	G2:11	CTCAE
**Petrongari et al**	2013 (39)	Prospective phase II	71 M	I:100	IMRT CV	86Gy/43(2)	[86Gy]	39(-)	G2:51	G2:44	G2:5;G3:8	G2:18;G3-G4:2,5%	CTCAE
**Wu et al**	2012 (73)	Prospective phase II	54 M	L:25;I:75	Hypo-IMRT IGRT (fiducials)	55 Gy/16(3.43)	[77.6Gy]	11(+) 62(-)	G2:41;G3:3	G2:34;G3:4	G2:8	G2:8	RTOG
**Zilli et al**	2011 (82)	Prospective randomized phase III	48 M	L:28; I:44;H:28	Hypo-IMRT	54Gy/14 (3.85)	[82.7Gy]	11(+) 71 (-)	G1:22;G2:4	G1:8;G2:4	G1:9;G2:5	G1:13;G2:3	RTOG
**Lock et al**	2011 (66)	Prospecitve phase II	36 M	L:27;I:36; H:1	Hypo-IMRT VMAT	63,2Gy/20(3.16)	[84.1Gy]	6(+) 60(-)	G1:51,5;G2:33,8;G3:8,8	G1:42,4;G2:25;G3:10,3; G4:1,5	G1:54,7;G2:14,1; G3:4,7	G1:39,1;G2:25;G3:3,1; G4:1,6.	CTCAE
**Martin et al**	2007 (92)	Prospecitve phase II	38 M	L:29;I:56; H:7	Hypo-IMRT	60Gy/20(3)	[77.1Gy]	8(+) 84(-)	G1:43;G2:25;G3:0	G1:22;G2:11;G3:1	G1:7;G2:3;G3:0	G1:2;G2:4;G3:0	RTOG
Randomized Studies for Meta-Analysis													
**Dearnaley et al**	2016 (1065)	Prospecitve randomized phase III	62.4 M	L:15;I:73; H:12	IMRT CV	74Gy/37(2)	[74Gy]	239(+)	G>2:46	G>2:25	G2:9.1; G>3:<1	G2:13.7; G>3:0	RTOG/ LENT
	(1077)				Hypo-IMRT	57Gy/19(3)	[73.3Gy]		G>2:46	G>2:38	G2:6.6; G>3:1	G2:11.3; G>3:<1	
	(1074)				Hypo-IMRT	60Gy/20(3)	[77.1Gy]		G>2:49	G>2:38	G2:11.7; G>3:<1	G2:13.7; G>3:<1	
**Pollack et al**	2006 (50)	Prospecitve randomized	3 M	I:65; H:35	IMRT CV	76Gy/38(2)	[76Gy]	44(+) 56(-)	G1:28;G2:54;G3:2	G1:40;G2:8;G3:0	G1:31;G2:8;G3:0	G1:12;G2:2;G3:0	RTOG/ LENT
	(50)				Hypo-IMRT	70.2Gy/26(2.7)	[84.2Gy]		G1:44;G2:40;G3:8	G1:40;G2:18;G3:0	G1:30;G2:6;G3:0	G1:16;G2:0;G3:0	
**Pollack et al**	2013 (153)	Prospecitve randomized	68.4 M	L-I:50;H:50	IMRT CV	76Gy/38(2)	[76Gy]	139(+)	G>2:5.2	G>2:22.5	G>2:14.6	G1:58.9;G2:20.5;G3:2	RTOG
	(154)				Hypo-IMRT	70.2Gy/26(2.7)	[84.2Gy]		G>2:10.6	G>2:18	G>2:15.3	G1:53.7;G2:16.1;G3:2	

Risk classes, technique used, total dose, type of fractionation, equivalent dose, acute and late gastrointestinal and genitourinary toxicity. Abbreviations: FU (Follow up); Risk groups: L (Low), I (Intermediate), H (High); CV (conventional); Hypo (Hypofractionated); IMRT (Intensity Modulated Radiation Therapy); PBT (Proton Beam Therapy); EQ (Equivalent Dose); CK (Cyberknife); EBRT (External Beam Radiation Therapy); IGRT (Image Guided Radiotherapy); GU (Genitourinary); GI (Gastrointestinal); ADT (androgen deprivation therapy).

**Table 3:** Summary of trials on extreme hypofractionated Stereotactic Body Radiation Therapy (SBRT) treatment

Author	Year (No.pt)	Type of Study	FU (median)	Risk groups (L/I/H)%	SBRT	RT(total dose/n.f) (daily Fx)	[EQ D2] a/b 1,5	ADT	Acute GU Toxicity %	Acute GI Toxicity %	Late GU Toxicity %	Late GI Toxicity %	Toxicity (Criteria)
**Rucinska et al.**	2016 (68)	Prospective	24 M	L:10;I:90	Linac	33,5/5 (6,7)	[78,5Gy]	16(-) 52(+)	G1:32,3;G2:35,3;G3:1,5	G1:26,5;G2:10,3	G1:41,2;G2:11,8	G1:17,6;G2:4,4	RTOG/EORTC
**Loblaw et al.**	2013 (84)	Prospective phase I/II	55 M	L:100	Linac	35Gy/5(7) weekly	[85.0Gy]	1(+)	G1:71;G2:19;G3:1	G1:67; G2:10; G3:1	G1:2;G2:5	G1:35; G2:7; G4:1	CTCAE/RTOG
**Madsen et al.**	2007 (40)	Prospective phase I/II	41 M	L:40	Linac	33,5Gy/5(6,7)	[78,5Gy]	/	G1:28;G2:20,5;G3:3	G1:26;G2:13	G1:25;G2:20	G1:30;G2:7,5	CTC
**Boike et al**	2011 (48)	Prospective	30 M	L:20 I:80	Linac	45Gy/5(9)	[135Gy]	(+)27% (-)73%	G1:20; G2:27	G1:40;G2:0	G1:20; G2:13; G3:	G1:7; G2:7; G4:0	CTCAE
				L:53 I:47		47,5Gy/5(9.5)	[149.3Gy]	(+)13% (-)87%	G1:33;G2:7	G1:13; G2:27	G1:20; G2:13; G3:7	G1:27; G2:7; G4:0	
				L:47 I: 53		50Gy/5(10)	[164.3Gy]	(+)27% (-)73%	G1:33;G233	G1:47; G2:47	G1: 0; G2: 0; G3: 7	G1:33; G2:0; G4:7	
**Fuller et al.**	2014 (79)	Prospective phase II	72 M	L:51;I:49	CK	38Gy/4 (9.5)	[119,4Gy]	(-)	G2:10	G1:0;G2:0	G2:9;G3:6	G2:1	CTCAE
**Bolzicco et al.**	2013 (100)	Prospective	36 M	L:41;I:42; H:17	CK	35Gy/5(7)	[85.0Gy]	29(+)	G1:34; G2:12	G1:27; G2:18	G1:4;G2:3	G1:2;G2:1	RTOG
**Katz et al.**	2013 (50)	Prospective	60 M	L:69; I:27; H:4	CK	35Gy/5(7)	[85.0Gy]	57(+)	G1:72;G2:4	G1:76;G2:4	G2:4	G2:2	RTOG
	(254)					36,25Gy/5(7,25)	[90,6Gy]		G1:74,8;G2:4,7	G1:74,4;G2:3,5	G2:9;G3:2	G2:5	
**King**	2012 (67)	Prospective phase II	31 M	L:100	CK	36,25Gy/5(7.25)	[90,6Gy]	67(-)	G1:23;G2:5;G3:3	G1:12,5;G2-3:0,2	G1:23; G2:5;G3:3,5	G1:14;G2:2	RTOG
**Bolzicco et al.**	2010 (45)	Prospective	20 M	L:49;I:51	CK	35Gy/5(7)	[85.0Gy]	17(+)	G1:35,5;G2:11,1	G1:24,4;G2:24,4	G1:8,8;G3:2,2	G2:2,2	RTOG
**King et al.**	2009 (41)	Prospective phaseII	33 M	L:100	CK	36,25Gy/5(7,25)	[90,6Gy]	(-)	G1:41;G2:24;G3:5	G1:33;G2:15	G1:14;G2:7;G3:3	G1:28;G2:19; G3:7	RTOG

Risk classes, technique used, total dose, type of fractionation, equivalent dose, acute and late gastrointestinal and genitourinary toxicity. Abbreviations: FU (Follow up); Risk groups: L (Low), I (Intermediate), H (High); EQ (Equivalent Dose); CK (Cyberknife); SBRT (Stereotactic Body Radiation Therapy); GU (Genitourinary); GI (Gastrointestinal); ADT (androgen deprivation therapy).

**Table 4:** Mean of percentage of toxicity G2 and > G3 in different type of studies

	Acute Toxicity G2		Late Toxicity G2		Acute Toxicity G3		Late Toxicity G3	
	GU	GI	GU	GI	GU	GI	GU	GI
**IMRT-Hypo (8 studies)**	31%	23%	9%	12%	7%	4%	3%	2%
**IMRT-CV (7 studies)**	31%	17%	11%	13%	2%	/	7%	2%
**3DCRT-Hypo (4 studies)**	22%	13%	6%	9%	5%	3%	2%	2%
**3DCRT-CV (3 studies)**	43%	41%	21%	18%	3%	2%	3%	2%
**PBT-CV (2 studies)**	13%	1%	16%	15%	/	/	6%	/
**SBRT-Linac (4 studies)**	24%	16%	13%	7%	2%	/	7%	/
**SBRT-CK (6 studies)**	10%	11%	6%	5%	4%	/	3%	/
**3DCRT + CK (2 studies)**	6%	7%	4%	/	/	/	/	/

Abbreviations: CV (conventional); Hypo (Hypofractionated); IMRT (Intensity Modulated Radiation Therapy); SBRT (Stereotactic Body Radiation Therapy); PBT (Proton Beam Therapy); CK (Cyberknife); GU (Genitourinary); GI (Gastrointestinal).

At a first analysis, the mean percentage of acute urinary and gastrointestinal G2 toxicity decreased in Hypo-3DCRT versus CV 3DCRT (22 and 13% vs 43% and 41% respectively), and in Hypo-IMRT versus CV 3DCRT (31 and 23% vs 43% and 41% respectively). There was an even greater reduction in eHYPO, especially with the Cyberknife system (10 and 11%). A reduction was also observed in the mean percentage of late urinary and gastrointestinal G2 and > G3 toxicity, in HYPO versus CV 3DCRT (6 and 9% vs 21 and 18% respectively), and with IMRT technique vs CV-3DCRT (9 and 12% vs 21 and 18% respectively). Even in SBRT treatment, there was a reduction of late urinary toxicity (6%) and late gastrointestinal toxicity (5%). Studies evaluating 3DCRT + CK showed a lower urinary and rectal toxicity. G3 toxicity presented values between 2-7% and wasn’t reported in all studies, preventing us to perform an appropriate analysis. Table 5 shows patients with acute/late genitourinary and gastrointestinal toxicity G0-G1 and > G2 for each study group.

**Table 5:** Number of patients with acute or late genitourinary and gastrointestinal toxicity G0-G1 and > G2

Groups of Studies			Acute GU Toxicity		Acute GI Toxicity		Late GU Toxicity		Late GI Toxicity	
		*N. total of pz*	*G0-1*	*>G2*	*G0-1*	*>G2*	*G0-1*	*>G2*	*G0-1*	*>G2*
**1**	IMRT Hypo	***2668***	1519	1148	1747	921	2402	266	2340	328
**2**	IMRT CV	***1794***	1123	671	1426	369	1591	202	1542	252
**3**	3DCRT Hypo	***990***	693	213	916	74	932	60	904	59
**4**	3DCRT CV	***1261***	805	388	1043	351	1062	199	1100	161
**5**	SBRT	***873***	682	124	798	73	791	82	827	45

Abbreviations: Hypo (Hypofractionated); IMRT (Intensity Modulated Radiation Therapy); SBRT (Stereotactic Body Radiation Therapy); GU (Genitourinary); GI (Gastrointestinal).

In Table 6 we summarized Odds Ratio (OR) results derived by comparing patients grouped according to different technology and fractionation schemes:

**Table 6:** Values of Odds Ratio (OR) of toxicity > G2 and G0-1 in the compared different groups of study

*Comparison of the groups*		*OR (95%CI)*	*p*
**1 vs 2** (IMRT Hypo vs IMRT CV)	**Acute GU**	**1.26 (1.12-1.43)**	**0.0002**
	**Acute GI**	**2.04 (1.77-2.34)**	**0.0000**
	Late GU	**0.87** (0.72-1.06)	0.1661
	Late GI	**0.86**(0.72-1.02)	0.0878
**3 vs 4** (3DCRT Hypo vs 3DCRT CV)	Acute GU	**0.64**(0.52-0.78)	0.0000
	Acute GI	**0.24**(0.18-0.31)	0.0000
	Late GU	**0.34**(0.25-0.46)	0.0000
	Late GI	**0.45**(0.33-0.61)	0.0000
**2 vs 4** (IMRT CV vs 3DCRT CV)	Acute GU	**1.24** (1.06-1.45)	0.0063
	Acute GI	**0.77** (0.65-0.91)	0.0020
	Late GU	**0.67** (0.54-0.82)	0.0001
	Late GI	**1.12** (0.90-1.38)	0.3087
**1 vs 3** (IMRT Hypo vs 3DCRT Hypo)	Acute GU	**2.46**(2.07-2.92)	0.0000
	Acute GI	**6.53**(5.08-8.38)	0.0000
	Late GU	**1.72**(1.29-2.30)	0.0002
	Late GI	**2.15**(1.61-2.86)	0.0000
**5 vs 1** (SBRT vs IMRT Hypo)	Acute GU	**0.24** (0.20-0.30)	0.0000
	Acute GI	**0.17** (0.13-0.22)	0.0000
	Late GU	**0.94** (0.72-1.21)	0.6190
	Late GI	**0.39** (0.28-0.54)	0.0000

Abbreviations: CV (conventional); Hypo (Hypofractionated); IMRT (Intensity Modulated Radiation Therapy); SBRT (Stereotactic Body Radiation Therapy); GU (Genitourinary); GI (Gastrointestinal); *p* (*p*-value).

From the analysis of OR values, it has been possible to postulated that:

The comparison between IMRT Hypo *vs* IMRT CV showed a not statistically significant lower risk in terms of G2 or worse late GU (*OR 0,87; p = 0,1661*) and GI toxicity (*OR 0.86; p = 0,0878*) for Hypo Group; on the other hand, HYPO Group seemed to be more exposed to G2 or worse acute GU (*OR 1.26 ; p = 0,0002*) and GI toxicity (*OR 2.04 ; p < 0,001*) than the conventional fractionation counterpart.

3DCRT Hypo showed a better safety profile for each anatomical district, both in early and late toxicity, than the traditional fractionation scheme.

3DCRT CV treated patients compared to IMRT CV counterparts seemed to be affected by a worse toxicity profile in terms of acute GI (*OR 0.77; p = 0.0020*) and late GU side effects(*OR 0.67; p = 0.0001*), while having a lower frequency of acute GU toxicity *(OR 1,24; p = 0,0063)*; no statistically significant difference was found for late GI toxicity.

IMRT Hypo showed an overall worse toxicity profile than the hypofractionated 3DCRT, with a 6,5 times higher frequency of acute GI side effects (*p < 0,001*).

Patients who underwent Stereotactic Radiotherapy (SBRT) have been affected to a lesser extent by acute GI and late gastrointestinal toxicity compared to patients treated with IMRT Hypo; no difference between these two study group was observed in terms of late genitourinary side effects, but in SBRT-group an higher risk of acute GU side effects was found.

Finally, in view of a not huge scientific soundness generated by comparing only single-arm, phase two studies data, we attempted to perform a meta-analysis involving IMRT groups, because, to date, randomized prospective trials have been carried out only with this technology.

**Figure 2:**
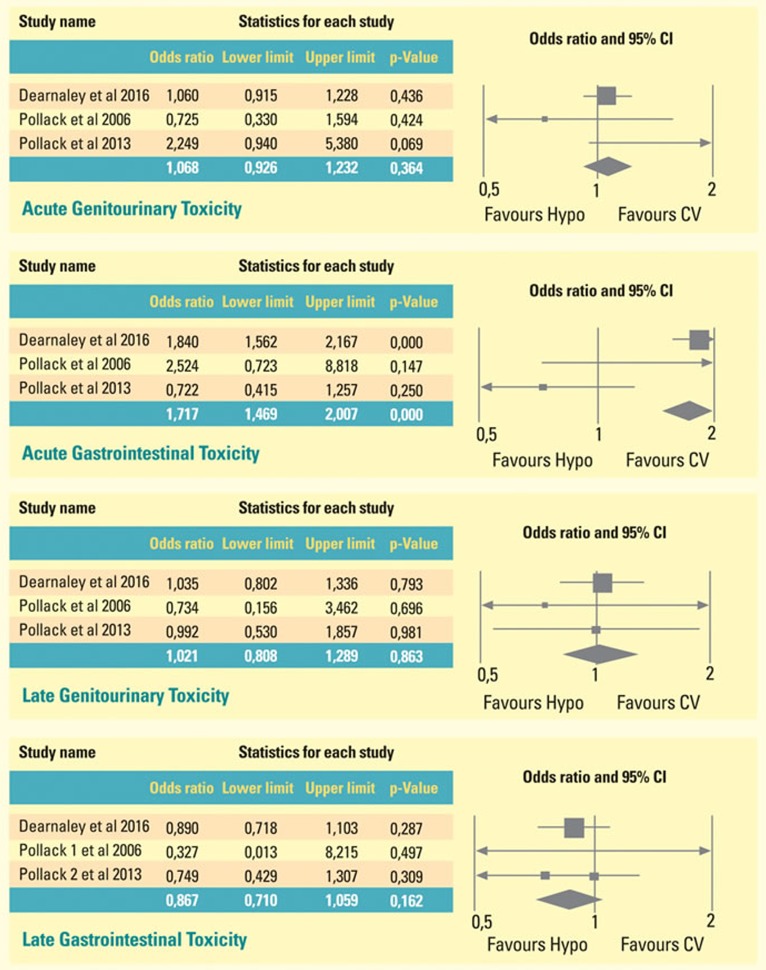
Meta-analysis of three randomized trials, which compare toxicities of patients treated with HYPO scheme with those treated with conventional fractionation Patients who underwent hypofractionated radiotherapy schemes suffered from GI acute toxicity to the extent of near 71% more than CV-treated counterparts (*p*-value < 0,001; Confidence Interval: 1,469- 2,007): conversely, Hypo-IMRT treated patients experienced less late gastrointestinal toxicity to the extent of 13 % than CV treated patients, without reaching statistical significance (OR 0,867 ; *p* = 0,162).

In Figure 2 we showed the meta-analysis of three randomized trials, which compare toxicities of patients treated with HYPO scheme with those treated with conventional fractionation. Patients who underwent Hypofractionated radiotherapy schemes suffered from GI acute toxicity to the extent of near 71% more than CV-treated counterparts (*p-value* < 0,001; *Confidence Interval: 1,469*- 2,007): conversely, Hypo-IMRT treated patients experienced less late gastrointestinal toxicity to the extent of 13 % than CV treated patients, without reaching statistical significance *(OR 0,867 ; p = 0,162)*

Certainly, this is a preliminary analysis, and the lack of homogeneous data and phase III studies strengths the need of prospective randomized trials, supporting us to really know the incidence of radiotherapy-induced rectal and urinary toxicity, which are serious issues affecting the quality of life and compliance to treatment of prostate cancer patients.

## DISCUSSION

The advent of image-based or image-guided RT, new therapeutical algorithms and technology advancement permitted the use of high-dose for fraction in prostate cancer treatment. Some tumors, such as prostate cancer, have a very low α/β ratio and higher single dose that can be applied with a better tumor control, without increasing side effects [21,22]. Randomized trials have shown a superior biochemical control when higher total doses of conventionally fractionated irradiation are delivered to prostate [23,24]. However, dose escalation with standard fractionation improves biochemical-free survival at the expense of an overall treatment duration, that is longer [10, 25–26].

Target localization prior to daily treatments is required and can be performed using X-ray imaging of implanted fiducial markers: this technique allows a smaller Planning Tumor Volume (PTV) expansion with a lower dose to the surrounding organs. The accuracy of different real-time localization systems can vary considerably: for example, with the Novalis or Varian True Beam systems, localization and target positioning before each treatment fraction is needed. With the Calypso system, the operator sets a threshold (typically 3-5mm) beyond which the treatment is interrupted and the patient correctly repositioned. With the CyberKnife, continuous image acquisition and target correction occur routinely: correction for target motion must account for translational (anterior/posterior, right/left, and superior/inferior) motion. Stereotactic body radiotherapy (SBRT) delivers a very high-dose radiotherapy to body targets, with the treatment accomplishment in one to five fractions. The Quantitative Analysis of Normal Tissue Radiation Effects in the Clinic (QUANTEC) emphasizes the importance of an adequate organ volume delineation to get radiation dose-volume parameters and OARs radiation tolerance constraints [27].

The rectal morbidity (such as proctitis, ulceration) is lower when less than the 25% of rectal volume receives doses of < 70Gy. Rectal bleeding occurred in 1% of patients when V_65_ was < 23% and increased to 10% with V_65_ ≥ 28%, because the *α/β ratio* of rectum is usually assumed to be 3 Gy, but it might be higher [28–30]. In a study of 101 patients with prostate cancer patients treated with 3D-CRT or IMRT (mean dose 70-74Gy) and evaluated with proctoscopy within 1 year after treatment, V_60_ and V_70_ were related to incidence of rectal telangiectasias and bleeding. No data on late rectal tolerance to hypofractionated stereotactic irradiation (36-40 cGy in five fractions) are available. The *α/β ratio* of a normal bladder is assumed to be in the region of 3-5 Gy for late toxic effects and near to 10 Gy for acute toxicity. The trigonal area appears to be very radio-sensitive, sometimes resulting in fibrosis-related urethral obstruction. Regard to the tolerance of the urinary bladder, equivalent dose at 80Gy for partial organ and at 50Gy for the whole have been established. Marks et al. estimated a clinical complication rate of 5-10% with 50 Gy given to the whole bladder in 2Gy fractions. Similar toxicity has been observed with 60-65Gy to partial bladder volumes. The RTOG 0415 study of prostate cancer patients included a bladder dose-volume constraint of no more than 15% of the volume to receive >80 Gy, no more than 25% of the volume to receive > 75 Gy, no more than 35% of the volume to receive >70 Gy, and no more than 50% of the volume to receive >65 Gy. Urethral tolerance has been estimated at 65-70Gy with 2Gy external irradiation [31–35]. In this work, the acute and late urinary and gastrointestinal toxicity detected in patients with low-intermediate risk localized prostate cancer were analyzed, also in relation to used technique and fractionation scheme. Schmid et al. [36] conducted a prospective phase II study of 178 primary prostate-cancer patients, including 123 patients with low/intermediate risk treated with a dose of 70 Gy and 55 patients treated with 74 Gy in conventional fractionation: G2 acute GI and GU toxicity were 15% and 22% respectively, while G2 late GI and GU toxicity were 19% and 23% respectively. Authors concluded that most of radio-induced late GI and GU side effects were transient.

Michalsky et al [37] evaluated 219 patients treated with 78 Gy in 39 fractions, 119 on prostate (first arm) and 100 patients treated both on prostate and seminal vescicles (second arm). The percentage of G2 acute urinary and rectal toxicity were 36% in the first arm, 45% in the second. Late GU toxicity G2 were 14% in the first arm and 20% in the second arm; the late GI toxicity were 19% in the first arm and 17% in the second arm.

Zietman et al [38] evaluated a total of 394 men, randomized to receive 50.4 Gy and a boost of 19.8 Gy protons in 11 fractions (first arm), or a boost of 28.8 Gy in 16 fractions: two percent of patients in both arms experienced late grade 3 genitourinary toxicity, 1% of patients in the high-dose arm experienced late grade 3 GI toxicity. Acute and late toxicity of grade 2 were 51 and 22% genitourinary and 44 and 13% gastrointestinal for low-dose arm; 60 and 27% genitourinary and 63 and 24% gastrointestinal for high-dose arm.

Two proton therapy with conventional fractionation trials were then evaluated: first trial, conducted by Coen et al. [39], with a dose of 78-79 Gy in 85 men, reported genitourinary/gastrointestinal acute toxicity of 51/19% Grade 1; of 13/1% Grade 2; of 1/0% Grade 3 respectively. Late genitourinary/gastrointestinal toxicity were of 33% Grade 1; 26% Grade 2; 11% Grade 3; 2% Grade 4. The second trial conducted by Nihei et al [40] with 50 Gy dose of Protons and boost of 24 Gy, reported genitourinary/gastrointestinal acute toxicity of 58/10% Grade 1; of 12/1% Grade2 respectively. Late genitourinary/gastrointestinal toxicity, were of 6/18% Grade 1; of 1/3% Grade 2 respectively.

In the first group of study with 3DCRT technique, five prospective studies with hypofractionated scheme were assessed. White et al [41] evaluated 90 patients treated with 57 Gy in 17 fz of 3Gy and have reported acute genitourinary toxicity G 1, 2 and 3 to 58.6%, 10% and 1.1% respectively; acute gastrointestinal toxicity to 75.6%, 9% and 0% respectively. Grade 1, 2 and 3 GU and GI late toxicity were 47.3%, 2.4%, 0%, and 40%, 9.3% and 4.7% respectively. Tramacere et al [42] evaluated 97 patients treated with a schedule of 62 Gy in 20 fractions over 5 weeks, 4 fractions of 3.1 Gy each for week, and reported genitourinary (GU) and gastrointestinal (GI) ≥ G2 acute toxicities of 21% and 15%, a late toxicity of 8% and 11% respectively. Jereczek-Fossa et al [43] evaluated 337 patients treated with 70 Gy in 28 fz of 2.5 Gy and reported a G2 urinary and rectal acute toxicity of 35% and 11.3%, a late toxicity of 10.4% and 7.5%. Martin et al [44] evaluated 92 patients treated with 60 Gy in 20 fz of 3Gy, reported a G2 acute urinary and rectal toxicity of 25% and 3%, a late urinary and rectal toxicity of 11% and 4%.

Lukka et al [45] randomized 936 patients into two treatment arms: 470 patients received 66 Gy in 33 fractions and 466 patients 52.5 Gy in 20 fractions. Acute toxicity was found to be slightly higher (11.4%) in the short arm compared to the long arm (7%). The late toxicity was similarly low in both arms (3.2%).

Two interesting studies have used 3DCRT technique, one with conventional fractionation and one with hypofractionated, followed by a SBRT-boost with Cyberknife system to 9.5-4.75/fz the first, 9-9.75/fz the second. The mean percentage of acute genitourinary and gastrointestinal toxicity was 5% and 7%; the mean percentage of late toxicity was 4% and 08% respectively [46,47].

Other research groups considered the use of IMRT technique: 4 prospective studies with conventional fractionation, 4 with hypofractionated schemes and 3 prospective randomized studies. In a prospective study with conventional fractionation, Fang et al [48] evaluated 394 patients treated with 79.2 Gy in 44 fractions of 1.8Gy (213 treated with IMRT photon, 181 with protons). The urinary and gastrointestinal acute G2 toxicity were 28.7% and 18.3% respectively in IMRT and 21.3% and 12.8% in PBT; G2 late toxicities were 13.8% and 10.8% in IMRT and 4.3% and 12.8% in PBT. The prospective study of Goineau et al [49] evaluated 38 patients treated with 76 Gy with conventional fractionation and reported acute urinary and gastrointestinal G2 toxicity of 5.3%, and late G2 toxicity of 5.3% and 15.8%. Marchand et al [50] assessed the same doses in 55 patients and reported acute urinary and gastrointestinal G2 toxicity of 38% and 15%, late G2 toxicities of 13% and 11%. The prospective study of Petrongari et al [51] evaluated 39 patients treated with 86 Gy dose escalation, in 43 fractions of 2 Gy. They recorded acute urinary and gastrointestinal G2 toxicity of 51% and 5%, and late G2 toxicities of 44% and 18%.

Wu et al [52] evaluated a dose/fraction of 3.43Gy for a total dose of 55Gy, Zilli et al [53] a dose/fraction of 3.85 Gy for a total dose of 54 Gy, Lock et al [54] a dose/fraction of 3.16Gy for a total dose of 63.2 Gy. They recorded acute urinary and gastrointestinal G2 toxicity of 41-8%; 22-2.5%; 33.8-14.1 % respectively, and late G2 toxicity of 34-8%; 4-3%; 25-25% respectively. Martin et al [55] evaluated 92 patients treated with hypofractionated RT with 60 Gy in 20 fractions. They recorded acute urinary and gastrointestinal G2 toxicity of 25% and 11%, late urinary and gastrointestinal G2 toxicity of 3% and 4%.

Dearnaley et al [56] in a prospective randomized trial and Pollack et al [57,58] in two studies compared the acute and late toxicity between the conventional fractionation arm and the hypofractionated one, all performed with IMRT. Dearnaley et al reported a greater genitourinary and rectal acute toxicity in the fractionation of 60Gy in 20 fz vs 74 Gy in 37 fz (49% and 38% vs 46% and 25%), while late toxicity frequencies were equivalent, but no significant difference in 5 years side-effects incidence after treatment was observed.

Pollack et al, in the first study reported a greater toxicity in the hypofractionated treatment group (70.2Gy in 26 fz vs 76Gy in 38fz) only for acute gastrointestinal (18% vs 8%), instead G2 recorded in the lower genitourinary acute toxicity (48% vs 56%), in the late genitourinary (6% vs 8%), and in late gastrointestinal (0% vs 2%). In the second study, Pollack et al, showed increased toxicity genitourinary acute and late in hypofractionated scheme (70.2 Gy in 26 fz vs 76 Gy in 38 fz), lower the gastrointestinal. The meta-analysis showed an advantage in terms of acute gastrointestinal and late genitourinary toxicity for Hypofractionated schemes.

Finally, 10 studies of extreme hypofractionated scheme with SBRT technique, 4 SBRT with Linac, 6 SBRT with Cyberknife system were evaluated. The first 4 have evaluated treatments with dose/fraction of 6.7- 10 Gy for a total dose 33.5-50 Gy and number of fractions between 4 and 5. The mean of percentage of acute urinary and rectal G2 toxicity was between 24% and 16%, the late G2 toxicity was between 13% and 7% [59–62]. Regarding the studies that evaluated treatment using SBRT technique with Cyberknife system, were carried out using a 7-9.5 Gy dose/fraction for a total dose of 35-38 Gy and number of fractions between 4-5. The mean percentage of acute urinary and rectal G2 toxicity was 10% and 11%; the late G2 toxicity was 6% and 5% [63–68].

## CONCLUSION

Our review suggests that hypofractionated schemes, which have a recognized radiobiological value, are usually characterized by a good tolerance to treatment. The new treatment systems combined with advanced technology, as well as SBRT with Cyberknife system, represent a promising approach in the radiation treatment of prostate cancer. Actually, our paper doesn’t want to establish a definitive truth: very few trials assessed only low-intermediate risk-class patients, and our purpose is to “turn on” the debate about Fractionation Schedules, in order to stimulate further randomized prospective trials focusing both on the effectiveness and on the toxicity profile (toxicity/effectiveness ratio), in view of rising costs resulting from the use of new technologies.

## MATERIALS AND METHODS

### Study selection

In July 2016 by using PubMed on-line database (http://www.ncbi.nlm.nhi.gov/pubmed), “rectal toxicity”, “urinary toxicity”, “radiotherapy” and “localized prostate cancer” were the searched terms, with no limitation on publication date. Duplicates, retrospective studies, brachytherapy, only methodology, dosimetry, old techniques, advanced disease, after-surgical treatment or high-risk patients studies were excluded. Prospective studies, concerning potential relationship between acute/late genitourinary (GU)/gastrointestinal (GI) toxicity and prostate radiotherapy in patients with low/intermediate risk localized prostate cancer, were included in the final analysis.

Data collected from single arm, phase II non randomized studies have been evaluated in order to perform OR for toxicity risk, by using SPSS 19 (IBM Software, Armonk, NY, USA, 2010).

Furthermore, we considered suitable for the metanalysis randomized prospective trials, that had recruited low/intermediate Prostate Cancer risk patients, with available data on ≥ G2 toxicity frequency: our choice fell on low/intermediate risk Prostate Cancer patients who didn’t undergo surgery, because our purpose was to evaluate a sample with a treatment volume as homogeneous as possible. Unfortunately, we found no trial with this risk-class patients only: therefore, we decided to include in our analysis studies with no more than 50% of high risk patients.

Notwithstanding Hazard Ratio for toxicity-free survival was the endpoint in selected studies, we collected only event data and sample size in each group to perform Odds Ratio: in fact, our purpose was to determine whether there was a frequency difference in G2 or worse toxicity between the Hypofractionated and Conventional Treatment group, despite the time-to-event variable.

### Data extraction and analysis of results

For each study first author name, year of publication, type of trial, median follow-up, risk class, RT protocol, total dose and equivalent dose, androgen deprivation therapy (ADT), Toxicity Criteria, percentage of acute and late genitourinary and gastrointestinal toxicity were considered. The studies have been combined according to the type of technique (3DCRT, IMRT, SBRT) and type of fractionation (CV, HYPO, eHYPO). The mean of the percentage for toxicity ≥ G2 in each group was then calculated. The studies were gathered into 5 groups (IMRT-Hypo; IMRT-CV; 3DCRT-Hypo; 3DCRT-CV and SBRT) and the detected acute and chronic toxicity frequency differences between groups were analyzed by calculating OR: in a dichotomous point-of-view, we chose a toxicity ≥ G2 as the outcome event variable, compared to G0-G1 toxicity as no event. Similarly, we performed a meta-analysis of randomized prospective studies meeting previously mentioned criteria by using Comprehensive Meta-Analysis software (Biostat 14 North Dean Street, Englewood, USA).

The 95% confidence interval was estimated, considering *p-values* ≤ 0.05 statistically significant.

## References

[1] Teh BS, Mai WY, Uhl BM, Augspurger ME, Grant WH, Lu HH, Woo SY, Carpenter LS, Chiu JK, Butler EB Intensity-Modulated Radiation Therapy (IMRT) for prostate cancer with the use of a rectal balloon for prostate immobilization: acute toxicity and dose volume analysis. Int J Radiat Oncol Biol Phys.

[2] De Langhe S, De Ruyck K, Ost P, Fonteyne V, Werbrouck J, De Meerleer G, De Neve W, Thierens H Acute radiation-induced nocturia in prostate cancer patients is associated with pretreatment symptoms, radical prostatectomy, and genetic markers in the TGFBI gene. Int L Radiat Oncol Biol.

[3] Franco R, Caraglia M, Facchini G, Abbruzzese A, Botti G (2011). The role of tissue microarray in the era of target-based agents.-. Expert Rev Anticancer Ther.

[4] De Cobelli O, Buonerba C, Terracciano D, Bottero D, Lucarelli G, Bove P, Altieri V, Coman I, Perdonà S, Facchini G, Berretta M, Di Lorenzo G, Grieco P (2015). Urotensin II receptor on preoperative biopsy is associated with upstaging and upgrading in prostate cancer. -Future Oncology.

[5] Boccellino M, Alaia C, Misso G, Cossu AM, Facchini G, Piscitelli R, Quagliuolo L, Caraglia M (2015). Gene interference strategies as a new tool for the treatment of prostate cancer.-. Endocrine.

[6] Proust-Lima C, Taylor JM, Secher S, Sandler H, Kestin L, Pickles T, Bae K, Allison R, Williams S (2011). Confirmation of a low α/β ratio for prostate cancer treated by external beam radiation therapy alone using a post-treatment repeated-measures model for PSA dynamics. Int J Radiat Oncol Biol Phys.

[7] Miralbell R, Roberts SA, Zubizarreta E, Hendry JH (2012). Dose fractionation sensitivity of prostate cancer deduced from radiotherapy outcomes of 5,969 patients in seven international institutional datasets: α/β = 1.4 (0.9-2.2) Gy. Int J Radiat Oncol Biol Phys.

[8] Dasu A, Toma-Dasu I (2012). Prostate alpha/beta revisited - an analysis of clinical results from 14 168 patients. Acta Oncol.

[9] Brenner DJ, Martinez AA, Edmundson GK, Mitchell C, Thames HD, Armour EP (2002). Direct evidence that prostate tumors show high sensitivity to fractionation (low alpha/beta ratio), similar to late-responding normal tissue. Int J Radiat Oncol Biol Phys.

[10] Kuban DA, Tucker SL, Dong L, Starkschall G, Huang EH, Cheung MR, Lee AK, Pollack A (2008). “Long-term results of the M. D. Anderson randomized dose-escalation trial for prostate cancer,”. International Journal of Radiation Oncology, Biology, Physics.

[11] Heemsbergen WD, Al-Mamgani A, Slot A, Dielwart MF, Lebesque JV (2014). “Long-term results of the Dutch randomized prostate cancer trial: impact of dose-escalation on local, biochemical, clinical failure, and survival,”. Radiotherapy and Oncology.

[12] Zietman AL, Bae K, Slater JD, Shipley WU, Efstathiou JA, Coen JJ, Bush DA, Lunt M, Spiegel DY, Skowronski R, Jabola BR, Rossi CJ (2010). “Randomized trial comparing conventional-dose with high-dose conformal radiation therapy in early-stage adenocarcinoma of the prostate: long term results from. Proton Radiation Oncology Group/American College Of Radiology 95-09,” Journal of Clinical Oncology.

[13] Beckendorf V, Guerif S, Le Prisé E, Cosset JM, Bougnoux A, Chauvet B, Salem N, Chapet O, Bourdain S, Bachaud JM, Maingon P, Hannoun-Levi JM, Malissard L (2011). “70Gy versus 80Gy in localized prostate cancer: 5-year results of GETUG 06 randomized trial,”. International Journal of Radiation Oncology, Biology, Physics.

[14] Dearnaley DP, Jovic G, Syndikus I, Khoo V, Cowan RA, Graham JD, Aird EG, Bottomley D, Huddart RA, Jose CC, Matthews JH, Millar JL, Murphy C (2014). “Escalated-dose versus control-dose conformal radiotherapy for prostate cancer: long-term results from the MRC RT01 randomised controlled trial,”. The Lancet Oncology.

[15] Pawlowski JM, Yang ES, Malcolm AW, Coffey CW, Ding GX (2010). “Reduction of dose delivered to organs at risk in prostate cancer via image-guided radiation therapy,”. International Journal of Radiation Oncology Biology Physics.

[16] Gray PJ, Paly JJ, Yeap BY, Sanda MG, Sandler HM, Michalski JM, Talcott JA, Coen JJ, Hamstra DA, Shipley WU, Hahn SM, Zietman AL, Bekelman JE (2013). “Patient-reported outcomes after 3-dimensional conformal, intensity-modulated, or proton beam radiotherapy for localized prostate cancer,”. Cancer.

[17] Sheets NC, Goldin GH, Meyer AM, Wu Y, Chang Y, Stürmer T, Holmes JA, Reeve BB, Godley PA, Carpenter WR, Chen RC (2012). “Intensity modulated radiation therapy, proton therapy, or conformal radiation therapy and morbidity and disease control in localized prostate cancer,”. The Journal of the American Medical Association.

[18] Yu JB, Soulos PR, Herrin J, Cramer LD, Potosky AL, Roberts KB, Gross CP (2013). “Proton versus intensity modulated radiotherapy for prostate cancer: patterns of care and early toxicity,”. Journal of the National Cancer Institute.

[19] Klotz L, Zhang L, Lam A, Nam R, Mamedov A, Loblaw A (2010). Clinical results of long-term follow-up of a large, active surveillance cohort with localized prostate cancer. J ClinOncol.

[20] Edge SB, Byrd DR, Compton CC, Fritz AG, Greene FL, Trotti A (2010). AJCC Cancer Staging Manual.

[21] Daşu A (2007). Is the α/β Value for Prostate Tumours Low Enough to be Safely Used in Clinical Trials?. Clinical Oncology.

[22] Miralbell R, Roberts SA, Zubizarreta E, Hendry JH (2012). Dose-Fractionation Sensitivity of Prostate Cancer Deduced From Radiotherapy Outcomes of 5,969 Patients in Seven International Institutional Datasets: α/β = 1.4(0.9-2.2). Gy. Int J Rad Oncol Biol Physics.

[23] Fowler JF (2010). 21 years of biologically effective dose. Br J Radiol.

[24] Ritter M Rationale, conduct, and outcome using hypofractionated radiotherapy in prostate cancer. Semin Radiat Oncol.

[25] Peeters ST, Heemsbergen WD, Koper PC, van Putten WL, Slot A, Dielwart MF, Bonfrer JM, Incrocci L, Lebesque JV Dose-response in radiotherapy for localized prostate cancer: Results of the Dutch multicenter randomized phase III trial comparing 68 Gy of radiotherapy with 78 Gy. J Clin Oncol.

[26] Zietman AL, DeSilvio ML, Slater JD, Rossi CJ, Miller DW, Adams JA, Shipley WU Comparison of conventional- dose vs high-dose conformal radiation therapy in clinically localized adenocarcinoma of the prostate: a randomized controlled trial. JAMA.

[27] Bentzen Søren M., Constine Louis S., Deasy Y Joseph O., Avi Eisbruch Z, Andrew Jackson X, Marks K Lawrence B., Ten Haken Randall K., X, Yorke Ellen D (2010). Ph.D.K Quantitative Analyses Of Normal Tissue Effects In The Clinic (Quantec): An Introduction To The Scientific Issues - Int. J. Radiation Oncology Biol. Phys.

[28] Pollack A, Zagars GK, Smith LG, Lee JJ, von Eschenbach AC, Antolak JA, Starkschall G, Rosen I (2000). Preliminary results of a randomized radiotherapy dose-escalation study comparing 70 Gy with 78 Gy for prostate cancer. J Clin Oncol.

[29] Peeters ST, Lebesque JV, Heemsbergen WD, van Putten WL, Slot A, Dielwart MF, Koper PC (2006). Localized volume effects for late rectal and anal toxicity after radiotherapy for prostate cancer. - Int J Radiat Oncol Biol Phys.

[30] Marzi S, Saracino B, Petrongari MG, Arcangeli S, Gomellini S, Arcangeli G, Benassi M, Landoni V (2009). Modeling of alpha/beta for late rectal toxicity from a randomized phase II study: conventional versus hypofractionated scheme for localized prostate cancer. J Exp Clin Cancer Res.

[31] Majewski W, Maciejewski B, Majewski S, Suwinski R, Miszczyk L, Tarnawski R (2004). Clinical radiobiology of stage T2-T3 bladder cancer. Int J Radiat Oncol Biol Phys.

[32] Heemsbergen WD, Al-Mangami A, Witte MG, van Herk M, Pos FJ, Lebesque JV (2010). Urinary obstruction in prostate cancer patients from the Dutch trial (68 Gy vs 78 Gy): relationships with local dose, acute effects, and baseline characteristics. Int J Radiat Oncol Biol Phys.

[33] King CR, Brooks JD, Gill H, Presti JC (2012). Long-termoutcomes from a prospective trial of stereotactic body radiotherapy for low-risk prostate cancer. Int J Radiat Oncol Biol Phys.

[34] Marks LB, Carroll PR, Dugan TC, Anscher MS.- (1995). The response of the urinary bladder, urethra, and ureter to radiation and chemotherapy. Int J Radiat Oncol Biol Phys.

[35] Radiation Therapy Oncology Group RTOG 0415 - A phase III randomized study of hypofractionated 3D-CRT/IMRT versus conventionally fractionated 3D-CRT/IMRT in patients with favorable-risk prostate cancer.

[36] Schmid M, Pötter R, Bombosch V, Sljivic S, Kirisits C, Dörr W, Goldner G (2012). Late gastrointestinal and urogenital side-effects after radiotherapy - Incidence and prevalence. Subgroup-analysis within the prospective Austrian-German phase II multicenter trial for localized prostate cancer. Radiotherapy and Oncology.

[37] Michalski JM, Winter K, Purdy JA, Parliament M, Wong H, Perez CA, Roach M, Bosch W, Cox JD, Zietman A, Bae K, Slater J, Shipley W, Efstathiou J, Coen J, Bush D, Lunt M, Spiegel D, Skowronski R, Jabola R, Rossi C (2005). Toxicity after three-dimensional radiotherapy for prostate cancer on RTOG 9406 dose Level V. Int J Radiat Oncol Biol Phys.

[38] (2010). Randomized Trial Comparing Conventional-Dose With High-Dose Conformal Radiation Therapy in Early-Stage Adenocarcinoma of the Prostate - Long-Term Results From Proton Radiation Oncology Group/American College of Radiology 95-09. Journal of Clinical Oncology.

[39] Coen J, Bae K, Zietman A, Patel B, Shipley W, Slater J, Rossi C (2011). Acute and Late Toxicity after dose escalation to 82 GyE using Conformal Proton Radiation for localized prostate cancer: initial report of American College of Radiology Phase II Study 03-12. Int. J. Radiation Oncology Biol. Phys.

[40] Nihei K, Ogino T, Onozawa M, Murayama S, Fuji H, Murakami M, Hishikawa Y (2011). Multi-Institutional Phase II Study of Proton beam therapy for organ-confined prostate cancer. Focusing on the incidence of late rectal toxicities. Int. J. Radiation Oncology Biol. Phys.

[41] White R, Woolf D, Li S, Alonzi R, Osler P, Hoskin P, Hughes R (2015). Hypofractionated radiotherapy for localized prostate cancer using three dimensional conformal radiotherapy technique: 3 years toxicity analysis. Indian Journal of Cancer.

[42] Tramacere F, Arcangeli S, Pignatelli A, Castagna R, Portaluri M (2015). Hypofractionated Dose Escalated 3D Conformal Radiotherapy for Prostate Cancer: Outcomes from a Mono-Institutional Phase II Study. Anticancer Research.

[43] Jereczek-Fossa BA, Santoro L, Zerini D, Fodor C, Vischioni B, Dispinzieri M, Bossi-Zanetti I, Gherardi F, Bonora M, Caputo M, Vavassori A, Cambria R, Garibaldi C (2013). Image guided hypofractionated radiotherapy and quality of life for localized prostate cancer: prospective longitudinal study in 337 patients. - J Urol.

[44] Martin J, Rosewall T, Bayley A, Bristow R, Chung P, Crook J, Gospodarowicz M, Mclean M, Me´ Nard C, Milosevic M, Warde P, Charles Catton C (2007). Phase II Trial of hypofractionated Image-Guided Intensity Modulated Radiotherapy for localized prostate adenocarcinoma. Int. J. Radiation Oncology Biol. Phys.

[45] Lukka H, Hayter C, Julian JA, Warde P, Morris WJ, Gospodarowicz M, Levine M, Sathya J, Choo R, Prichard H, Brundage M, Kwan W (2005). Randomized trial comparing two fractionation schedules for patients with localized prostate cancer. - J Clin Oncol.

[46] Pontoriero A, Iatì G, Mondello S, Midili F, Siragusa C, Brogna A, Ielo I, Anastasi G, Magno C, Pergolizzi S, De Renzis C (2016). High-Dose Robotic Stereotactic Body Radiotherapy in the Treatment of Patients With Prostate Cancer: Preliminary Results in 26 Patients. Technology in Cancer Research & Treatment.

[47] Katz AJ, Santoro M, Ashley R, Diblasio F, Witten M (2010). Stereotactic Body Radiotherapy as Boost for Organ-confined Prostate Cancer. Technology in Cancer Research and Treatment.

[48] Fang P, Mick R, Deville C, Both S, Bekelman J, Christodouleas J, Guzzo T, Tochner Z, Hahn S, Vapiwala N (2015). A Case-Matched Study of Toxicity Outcomes After Proton Therapy and Intensity-Modulated Radiation Therapy for Prostate Cancer. Cancer.

[49] Goineau A, Marchand V, Rigaud J, Bourdin S, Rio E, Campion L, Bonnaud-Antignac A, Mahé M, Supiot S (2013). Prospective evaluation of quality of life 54 months after high-dose intensity- modulated radiotherapy for localized prostate cancer. Radiation Oncology.

[50] Marchand V, Bourdin S, Charbonnel C, Rio E, Munos C, Campion L, Bonnaudantignac A, Lisbona A, Mahe´ M, Supiot S (2010). No Impairment of quality of life 18 months after high-dose Intensity-Modulated Radiotherapy for localized prostate cancer: A Prospective Study. Int. J. Radiation Oncology Biol. Phys.

[51] Petrongari MG, Landoni V, Saracino B, Gomellini S, Arcangeli S, Iaccarino G, Pinnarò P, Arcangeli G, Strigari L (2013). Dose escalation using ultra-high dose IMRT in intermediate risk prostate cancer without androgen deprivation therapy: preliminary results of toxicity and biochemical control. Journal of Experimental & Clinical Cancer Research.

[52] Wu J, Brasher P, El-Gayed A, Pervez N, Tai P, Robinson J, Skarsgard D, Joseph K, Sia M, Pearcey R (2012). Phase II study of hypofractionated image-guided radiotherapy for localized prostate cancer: Outcomes of 55 Gy in 16 fractions at 3.4 Gy per fraction. Radiotherapy and Oncology 103.

[53] Zilli T, Jorcano S, Rouzaud M, Dipasquale G, Nouet P, Toscas J, Casanova N, Wang H, Escude´L Molla` M, Linero D, Weber D, Miralbell (2011). Twice-Weekly Hypofractionated Intensity-Modulated Radiotherapy for localized prostate cancer with low-risk nodal involvement: Toxicity and outcome from a dose escalation Pilot Study. Int. J. Radiation Oncology Biol. Phys.

[54] Lock M, Best L, Wong E, Bauman G, D’souza D, Venkatesan V, Sexton T, Ahmad B, Izawa J, Rodrigues G (2011). A Phase II Trial of Arc-Based Hypofractionated Intensity-Modulated Radiotherapy in localized prostate cancer. Int. J. Radiation Oncology Biol. Phys.

[55] Martin JM, Rosewall T, Bayley A, Bristow R, Chung P, Crook J, Gospodarowicz M, McLean M, Ménard C, Milosevic M, Warde P, Catton C (2007). Phase II trial of hypofractionated image-guided intensity-modulated radiotherapy for localized prostate adenocarcinoma -. Int J Radiat Oncol Biol Phys.

[56] Dearnaley D, Syndikus I, Mossop H, Khoo V, Birtle A, Bloomfield D, Graham J, Kirkbride P, Logue J, Malik Z, Money-Kyrle J, O’Sullivan JM, Panades M (2016). Conventional versus hypofractionated high-dose intensity-modulated radiotherapy for prostate cancer: 5-year outcomes of the randomised, non-inferiority, phase 3 CHHiP trial. - Lancet Oncol.

[57] Pollack A, Hanlon AL, Horwitz EM, Feigenberg SJ, Konski AA, Movsas B, Greenberg RE, Uzzo RG, Ma CM, McNeeley SW, Buyyounouski MK, Price RA (2006). Dosimetry and preliminary acute toxicity in the first 100 men treated for prostate cancer on a randomized hypofractionation dose escalation trial. - Int J Radiat Oncol Biol Phys.

[58] Pollack A, Walker G, Horwitz EM, Price R, Feigenberg S, Konski AA, Stoyanova R, Movsas B, Greenberg RE, Uzzo RG, Ma C, Buyyounouski MK (2013). Randomized trial of hypofractionated external-beam radiotherapy for prostate cancer. - J Clin Oncol.

[59] Rucinska M, Kieszkowska-Grudny A, Nawrocki S (2016). SHARP hypofractionated stereotactic radiotherapy is well tolerated in prostate cancer: Toxicity and quality of life assessment. Strahlenther Onkol.

[60] Loblaw A, Cheung P, D’Alimonte L, Deabreu A, Mamedov A, Zhang L, Tang C, Quon H, Jain S, Pang G, Nam R (2013). Prostate stereotactic ablative body radiotherapy using a standard linear accelerator: toxicity, biochemical, and pathological outcomes. Radiother Oncol.

[61] Madsen BL, Hsi RA, Pham HT, Fowler JF, Esagui L, Corman J (2007). Stereotactic hypofractionated accurate radiotherapy of the prostate (SHARP), 33.5Gy in five fractions for localized disease: first clinical trial results.Int. J Radiat Oncol Biol Phys.

[62] Boike TP, Lotan Y, Cho LC, Brindle J, DeRose P, Xie XJ, Yan J, Foster R, Pistenmaa D, Perkins A, Cooley S, Timmerman R (2011). Phase I dose-escalation study of stereotactic body radiation therapy for low- and intermediate-risk prostate cancer. J Clin Oncol.

[63] Fuller DB, Naitoh J, Mardirossian G (2014). Virtual HDR CyberKnife SBRT for Localized Prostatic Carcinoma: 5-Year Disease-Free Survival and Toxicity Observations. Front Oncol.

[64] Bolzicco G, Favretto MS, Satariano N, Scremin E, Tambone C, Tasca A (2013). A single-center study of 100 consecutive patients with localized prostate cancer treated with stereotactic body radiotherapy. BMC Urol.

[65] Katz AJ, Santoro M, Diblasio F, Ashley R (2013). Stereotactic body radiotherapy for localized prostate cancer: disease control and quality of life at 6 years. Radiat Oncol.

[66] King CR, Brooks JD, Gill H, Presti JC (2012). Long-term outcomes from a prospective trial of stereotactic body radiotherapy for low-risk prostate cancer. Int J Radiat Oncol Biol Phys.

[67] Bolzicco G, Favretto MS, Scremin E, Tambone C, Tasca A, Guglielmi R (2010). Image-guided stereotactic body radiation therapy for clinically localizedprostate cancer: preliminary clinical results. Technol Cancer Res Treat.

[68] King CR, Brooks JD, Gill H, Pawlicki T, Cotrutz C, Presti JC (2009). Stereotactic body radiotherapy for localized prostate cancer: interim results of a prospective phase II clinical trial. Int J Radiat Oncol Biol Phys.

